# Aggressive angiomyxoma as a rare cause of scrotum enlargement in a 10-month-old boy: a case report

**DOI:** 10.1186/s13256-022-03497-2

**Published:** 2022-07-30

**Authors:** Léonidas Nyandwi, Salahoudine Idrissa, Hellé Moustapha, Mahamoud Omid Ali Ada, Efared Boubacar, Idrissa Boubacar, Zakhama Abdelfatteh, Ksia Amine, Abarchi Habibou

**Affiliations:** 1 Amirou Boubacar Diallo National Hospital of Niamey, Niamey, Niger; 2 National Hospital of Niamey, Niamey, Niger; 3 Fattouma Bourguiba University Hospital of Monastir, Monastir, Tunisia

**Keywords:** Aggressive angiomyxoma, Scrotal mass, Infancy, Surgery, Case report

## Abstract

**Background:**

Aggressive angiomyxoma (AAM) is a locally infiltrative mesenchymal tumour that most commonly affects the pelvis and/or perineum in adult women. AAM is very rare in males, especially in infancy.

**Case presentation:**

A 10-month-old fulani (African) male infant was referred to our department for a large painless mass in the right testicule. The mass was detected during the neonatal period and gradually increased in size. Ultrasound examination revealed a large heterogeneous lesion; computed tomography results led to the conclusion that the mass was a mesenteric hernia. An inguinal and scrotal surgical approach was adopted. Exploratory surgery found a normal right testicle displaced upwardly and a large scrotal mass. Radical excision of the mass and orchidopexy were performed. Subsequent histology and immunohistochemstry studies indicated that the mass was a scrotal angiomyxoma. The postoperative course was uneventful. No recurrence occurred during the 6-month follow-up**.**

**Conclusion:**

To the best of our knowledge, this is the youngest patient with AAM reported to date. Angiomyxoma should be included in the differential diagnosis of scrotal masses, for which radical excision is justifiable to prevent recurrence.

## Introduction

Aggressive angiomyxoma (AAM) is a rare benign myxoid mesenchymal tumour. It is a non-metastasizing soft tissue tumour of the pelvis and perineum and occurs almost exclusively in adult females [1, 2], with only rare reports of AAM in males or involving the scrotum. Scrotal AAM mimics common paediatric pathologies, including hernia or hydrocele [2]. The recommended treatment for symptomatic patients is wide excision with tumour-free margins and close postoperative monitoring [3, 4]. Here, we report a case of AAM in a 10-month-old male who presented with voluminous slow-growing scrotal swelling.

## Case report

**Fig. 1:**
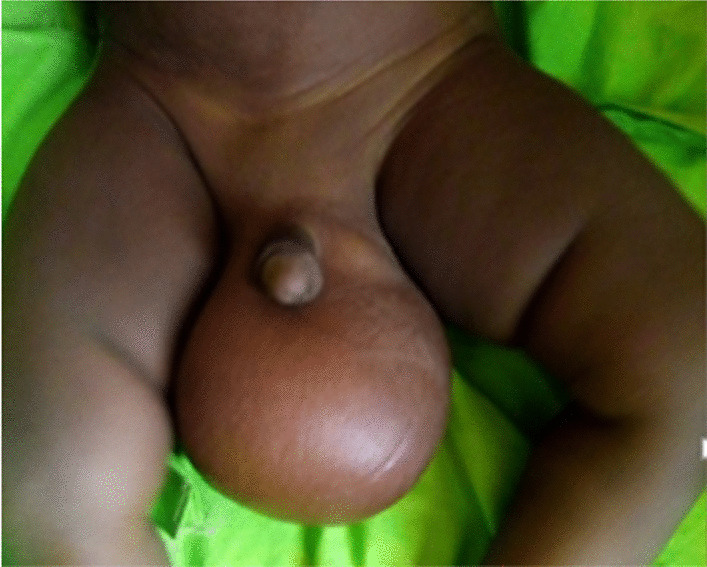
Preoperative appearance of a 10-month-old infant with scrotal angiomyxoma

A 10-month-old fulani (African) male infant was referred to our paediatric surgery department for a scrotal mass. The mass was first detected during the neonatal period and gradually increased in size. The infant had no known medical, surgical or family history. Clinical examination revealed an infant in good general condition, good nutritional status and an afebrile state with a voluminous right scrotal mass that was painless, spherical, soft, regular in shape and 10 cm in diameter, with some collateral venous circulation. A mass effect on the penis and perineum could be observed (Fig. 1). There was no transillumination. The right testicle was not found, and the left testicle was in the testicular bursa and appeared to be healthy. The clinical impression was initially oriented towards a testicular tumour diagnosis. Ultrasound examination revealed a large structural mass, with the heterogeneous echo measuring 10 × 7 × 6 cm. The two testes were upwardly displaced by the mass. Abdominopelvic computed tomography (CT) demonstrated a large intrascrotal tissue mass communicating with the abdomen with no visible air component, leading to the decision that the mass was probably an inguinal mesenteric hernia.

**Fig. 2:**
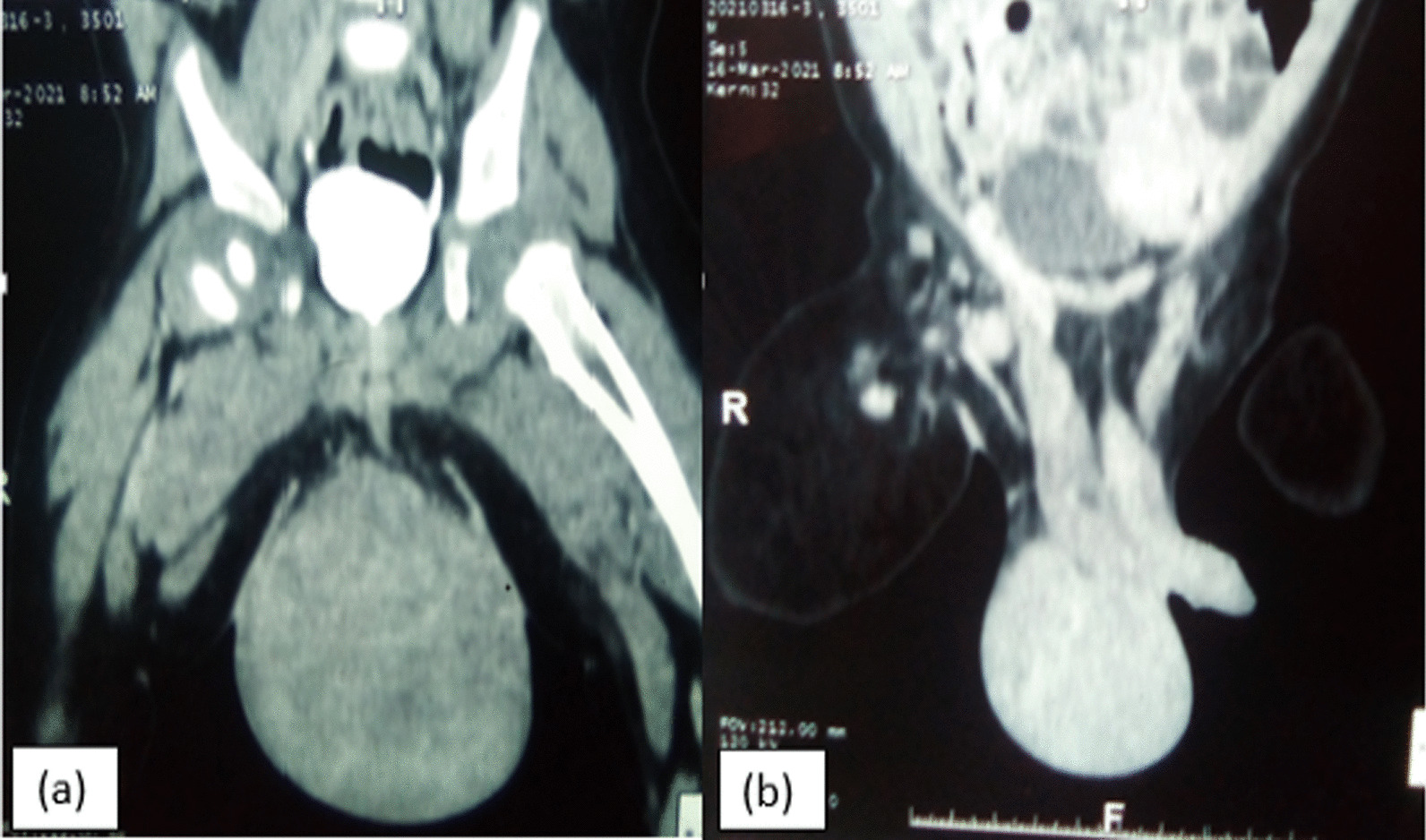
Computer tomography scan of the scrotal mass in bone (**a**) and parenchymal (**b**) windows

**Fig. 3:**
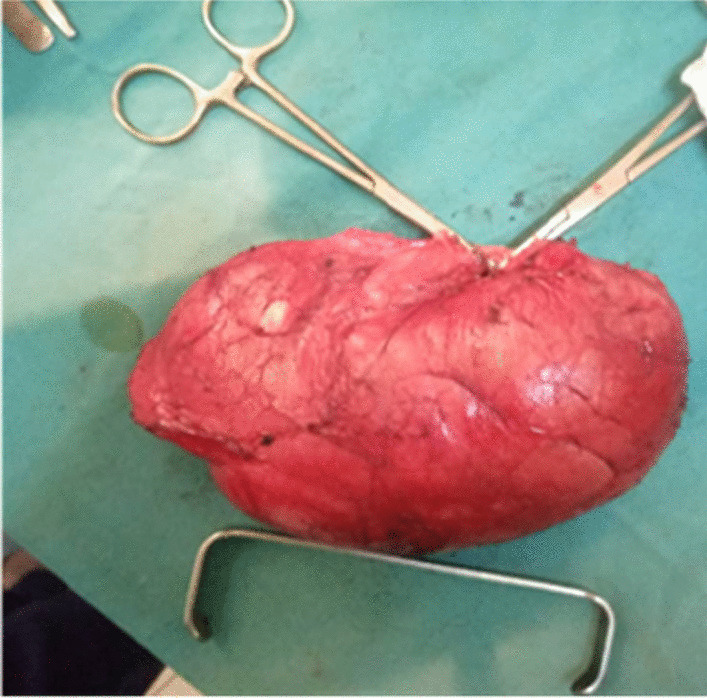
Postoperative appearance of the large mass

Serum tumour biomarkers, including α-fetoprotein and β-human chorionic gonadotropin levels, were all within normal limits. Surgery for the testicular tumour was proposed. Two combined surgical incision approaches were adopted, in which the right inguinal approach made it possible to locate the healthy right testicle in the inguino-scrotal region. The scrotal route was chosen for wide excision. During surgery, a vascular mass that displaced the testis upwards was found between the spermatic fascia and the skin (Fig. 2). The boundary of the tumour was not well circumscribed, and the originating organ of the tumour could not be determined. Scrotoplasty and right orchidopexy were performed. The dimensions of the lump were 11 × 9.5 × 8.5 cm and it weighed 400 g. The lump had two different appearances: one portion had the appearance of a typical solid tumour and the other showed mixed tumour growth. Microscopy examination revealed that the tumour had the histological appearance of a benign fibroblastic tumour, which was initially suggestive of angiomyxoma (Fig. 3a, b); the absence of malignancy suggested the need for an immunohistochemistry study to support this diagnosis.

**Fig. 4:**
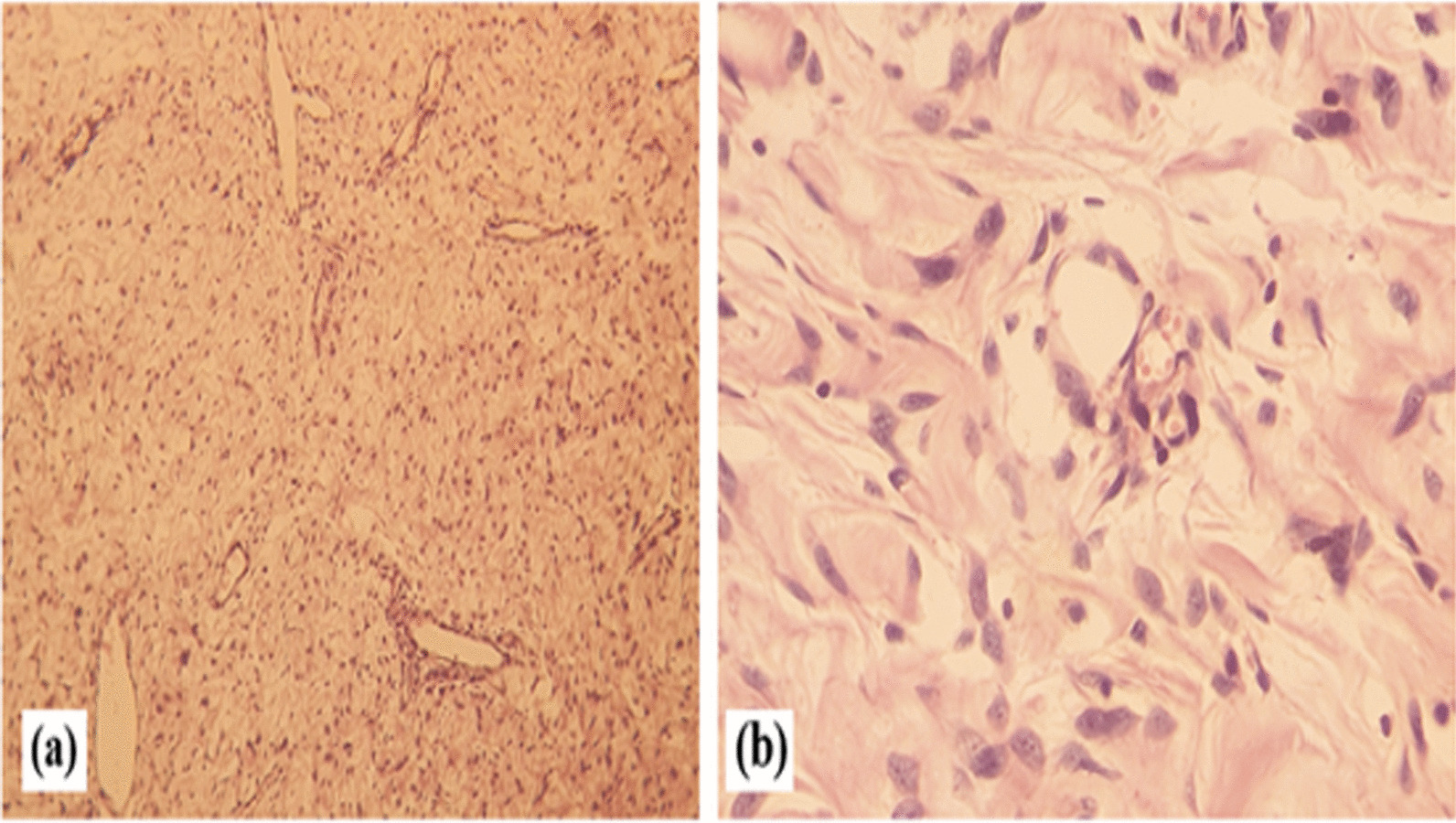
Histological features of angiomyxoma. **a** Microscopic image showing diffuse paucicellular proliferation with prominent vessels on a myxoid background (haematoxylin and eosin staining, magnification: ×50), **b** higher magnification showing spindle cells with oval nuclei without atypia (haematoxylin & eosin staining, magnification: ×400)

**Fig. 5:**
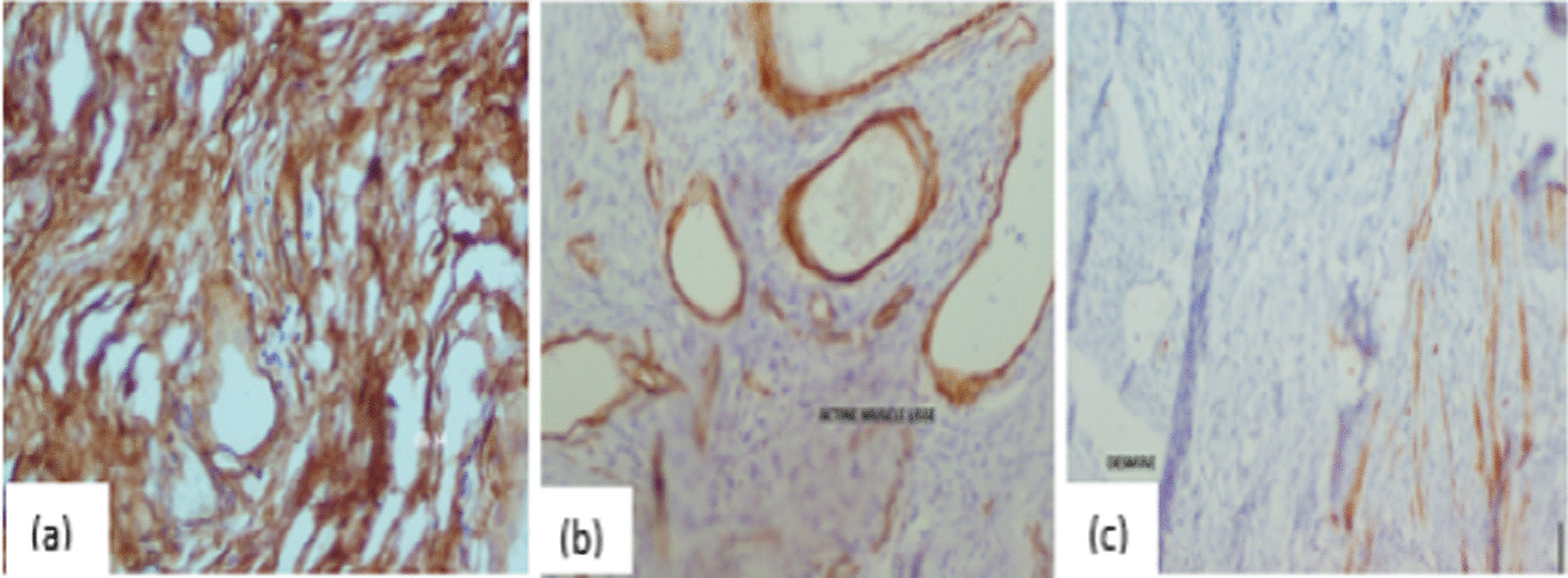
Immunohistochemical study findings. **a** Neoplastic cells with positive CD34 immunoreactivity, **b** actin staining, observed in the walls of the vessels and bundles of smooth muscle cells encompassing the tumour, **c** desmin staining, observed in the bundles of smooth muscle cells

Immunohistochemical staining for CD34, smooth muscle actin, desmin, myogenin and protein S was performed, with positive staining found only for CD34 in the tumor (Fig. 4a). Actin staining was seen in the walls of the vessels and bundles of smooth muscle cells encompassing the tumour (Fig. 4b); desmin staining was observed in the bundles of smooth muscle cells (Fig. 4c). Staining for the other markers was negative. This study confirmed the diagnosis of benign scrotal angiomyxoma and ruled out cell-based fusiform rhabdomyosarcoma. There were no postoperative surgical complications. No further recurrence was identified in the 6 months following surgery (Fig. 5).

## Discussion

Aggressive angiomyxoma is a rare tumour that is locally infiltrative but non-metastasizing. It occurs nearly exclusively in adult women of childbearing age and almost always arises in the perineum and pelvic area [5], occurring only rarely in other sites, such as the vagina, urinary bladder, various cutaneous locations, soft tissue of the perineum and perianal region, or in the uterine cavity as a polyp [1, 5, 6]. In males, AAM involves analogous sites, usually the scrotum[7, 8]. In our case, the tumour was located in the right scrotum. AAM is extremely rare in childhood [9]. To the best of our knowledge, the present case is the eighth to be reported in children, and it describes the youngest patient reported with AAM. Patients usually present with a long history of disease due to the slow growth of the tumour, without a history of pain, injury, fever or urinary tract symptoms. In general, AAM does not lead to distal metastases, and malignant transformation has not been observed [10]. During physical examination, AAM can mimic other common conditions causing scrotal enlargement; the tumour may present as a scrotal mass, which is often mistaken for a hernia or hydrocele [2, 4]. In our case, based on the results of the CT examination, we concluded that the mass was a mesenteric hernia.

 Tumour markers (alpha-fetoprotein, beta-human chorionic gonadotropin) and hormone levels (testosterone) contribute to the differential diagnosis and management of testicular masses in boys [11]. A few reported cases have included preoperative diagnoses based on histology. However, in most of the cases described to date, the diagnosis was made postoperatively following histological examination of the mass [4]. The diagnosis and distinction of AAM from other benign and malignant myxoid soft tissue tumours is mainly based on morphology and the exclusion of mimicking lesions with the help of immunohistochemistry [10]. Immunohistochemically, the cells expressed vimentin and, especially in peri-genital tumours, were positive for smooth muscle actin; in 50% of the tumours, CD34 positivity and S-100 negativity were observed. Most tumours showed positive oestrogen and progesterone expression, which expands the therapeutic options [11]. Our immunohistochemistry results were generally compatible with those reported previously. 

AAM must be differentiated from other benign myxoid neoplasms given its propensity for local recurrence, such as intramuscular myxoma, myxoid neurofibroma, neurothekeoma, spindle cell lipoma, superficial angiomyxoma, angiomyofibroblastoma, angiomyxolipoma and benign mixed mesenchymal tumours [12]. The more effective treatment for AAM is radical surgical excision that leads to free margins. Radiation therapy has been proposed to control multiple recurrences after surgical excision but has led to poor results; chemotherapy has also been shown to lead to poor results in AAM because of the slow progression of the disease. Incomplete excision almost always results in locoregional recurrence [4]. In the present case, no recurrence was found at the 6-month follow-up. Because late recurrence is possible, close follow-up remains necessary [4, 10].

## Conclusion

Aggressive angiomyxoma is a very rare benign neoplasm that is predominantly found in adult women. The present case describes the youngest male patient with AAM to date. Clinical examination and histology and immunochemistry studies were the main bases for the diagnosis of scrotal AAM, for which wide excision is justifiable to prevent recurrence.

## Data Availability

All data generated or analysed during this study are included in this published article.
